# A lighthearted approach to mindfulness: development and evaluation of a humor-enriched mindfulness-based program in a randomized trial

**DOI:** 10.3389/fpsyg.2023.1324329

**Published:** 2024-02-06

**Authors:** Christian T. Kastner

**Affiliations:** Applied Social and Health Psychology, Department of Psychology, University of Zurich, Zurich, Switzerland

**Keywords:** mindfulness, humor, well-being, stress, comic styles, gelotophobia, mindfulness-based intervention, randomized controlled trial

## Abstract

**Objectives:**

Recently, research on mindfulness and humor has begun to spark interest, both being essential contributors to well-being. This article proposes that forms of humor that share intention and attitudinal foundations with mindfulness, constitute the foundation of a mindful-humorous perspective and mindful humor. Once intention and attitude are in alignment, shared mechanisms underlying a mindful-humorous perspective may lead to synergetic effects (e.g., reinforcing a shift in perspective and attitudinal foundations) as summarized in the proposed mindful humor filter model (MHFM). Based on this theoretical framework, the humor-enriched mindfulness-based program (HEMBP) was developed and evaluated for validity and efficacy in a randomized controlled trial as a first step to test the newly introduced model.

**Methods:**

A total of 60 participants were randomly allocated to either the HEMBP or a wait-list control group. Participants' mindfulness, comic styles (e.g., benevolent humor, sarcasm, and cynicism), psychological well-being, life satisfaction, stress, and gelotophobia (fear of being laughed at) were assessed 1 week before and after training, and at a 1-month follow-up. Linear mixed-effects models were fitted to model changes in outcome variables over time.

**Results:**

The HEMBP was effective in increasing mindfulness, benevolent humor, and psychological well-being, and in reducing sarcasm, cynicism, stress, and gelotophobia, compared to the control group.

**Conclusion:**

Results support the validity and efficacy of the HEMBP as a promising approach for improving well-being and reducing stress. The HEMBP broadens the scope of existing mindfulness-based programs by cultivating a mindful-humorous outlook on life, facilitating mindfulness practice and insights, and fostering positive emotions and relationships with others through mindful humor. Results are also suggestive of the validity of the mindful humor filter model.

## 1 Introduction

Mindfulness and humor represent two invaluable, universal human capacities that are perhaps indispensable for living a good life. Mindfulness has only recently been (re)discovered in the West, despite having been cultivated for centuries as a path to alleviate suffering, which is ultimately a path to well-being (Hanh, 1998). Humor, specifically when not intended to harm others, has been praised as a crucial characteristic of a mature and healthy personality (Martin, 2003) by philosophers such as Aristotle (Chase, 1890) and psychologists like Freud (Freud, 1928), Maslow (Maslow, 1954), and Allport (Allport, 1961). While both qualities are important in their own right, the combination of their benefits might be more than the sum of their parts.

This article proposes that humor that shares a non-harming intention and attitudinal foundations (e.g., openness, acceptance, interest, and friendliness) with mindfulness (Schmidt-Hidding, 1963; Kabat-Zinn, 2011; Shapiro and Carlson, 2017) is the basis for a mindful-humorous perspective on life and mindful interpersonal humor. Such a perspective may yield synergetic effects, facilitating continuous mindfulness practice and the development of insights, wisdom, and compassion, as well as fostering positive emotions and relationships. Given their interconnected nature through intention, attention, and attitude (Shapiro et al., 2006), the cultivation of mindfulness might promote a mindful-humorous perspective. As such, both could be seamlessly integrated into one unified training.

The idea of combining mindfulness and humor is not new. The 14^th^ Dalai Lama is widely known for his good-natured humor, and renowned meditation teachers use humor in their teachings (e.g., Goenka, 1987; Goldstein, 1994; Rosenberg, 2004). Until now, however, there have been no systematic attempts to integrate humor into a standardized mindfulness-based program. It therefore seemed only natural to explore whether these two qualities could be integrated in a way “to navigate life's ups and downs—what Zorba the Greek called ‘the full catastrophe'—with grace, a sense of humor, and perhaps some understanding of the big picture, what I like to think of as wisdom” (Kabat-Zinn, 1990, p. 5).

This article has two main aims. First, the theoretical framework connecting mindfulness and humor will be delineated, summarized in the mindful humor filter model (MHFM). This model provides the basis for the structure and content of a newly designed humor-enriched mindfulness-based program (HEMBP). Second, the results of a randomized controlled trial testing the HEMBP for validity (its effects on mindfulness and different forms of humor) and efficacy (its effects on psychological well-being, life satisfaction, stress, and gelotophobia) will be presented.

The remainder of this introduction presents an overview of mindfulness and humor before considering which forms of humor might align with mindfulness. With this understanding, the potential added value of a mindful-humorous perspective and mindful humor will be explored. Lastly, the mindful humor filter model will be presented.

### 1.1 Mindfulness

The topic of mindfulness has seen a considerable increase in popularity in recent decades (Williams and Kabat-Zinn, 2011). Most contemporary definitions of mindfulness include an attention and attitude component, though Shapiro et al. (2006) highlight the importance of intention, or why one practices mindfulness. It is essential for mindfulness practice that one's intentions are non-harming (Kabat-Zinn, 2011; Shapiro and Carlson, 2017). The attitude component includes “an affectionate, compassionate quality within the attending, a sense of openhearted, friendly presence and interest” (Kabat-Zinn, 2003, p. 146), suggesting mindfulness is as much a quality of the heart as of the mind and a synonym for “heartfulness” (Kabat-Zinn, 2017). Intention, attention, and attitude represent three interwoven and interchanging aspects of mindfulness (Shapiro et al., 2006). Thus, mindfulness is operationally defined as a particular quality of intentional, continuous attention, characterized by an open, accepting, interested, and friendly attitude to present moment experiences.

A large body of literature corroborates the positive effects of mindfulness-based programs (MBPs; Crane et al., 2017) on a broad range of psychological (e.g., well-being and stress) and physical outcomes (e.g., pain) in healthy and clinical populations (Goldberg et al., 2022). MBPs typically comprise mindfulness-based stress reduction (MBSR; Kabat-Zinn, 1990) and mindfulness-based cognitive therapy (MBCT; Segal et al., 2013). The underlying assumption of MBPs is that intensive and systematic training in mindfulness leads to increased levels of mindfulness which then leads to positive outcomes, and there is moderate evidence that an increase in mindfulness does indeed mediate positive outcomes (Gu et al., 2015). However, starting mindfulness practice can be a challenge. Due to the nature of the human mind, hindrances such as mind-wandering or restlessness sooner or later arise, which the mind often tends to judge as negative or personal failures (Kabat-Zinn, 1990). Next to adapting MBPs to new populations and contexts (e.g., maximizing accessibility), MBPs that may facilitate starting and sustaining one's own mindfulness practice may provide additional benefits beyond existing MBPs (Loucks et al., 2022).

By intentionally inviting an open, accepting, interested, and friendly attitude toward present-moment experiences, participants of MBPs can learn to relate in a new way to those experiences, leading to a shift in perspective. This core mechanism of mindfulness has been termed reperceiving (Shapiro et al., 2006) or decentering (Segal et al., 2013), enabling participants to distance and disidentify themselves from those experiences. While focus is often placed on the attentional component of mindfulness, fostering a benevolent, loving-kind attitude toward present-moment experiences might be just as important (Grossman, 2015). This benevolent attitude could be complemented with a mindful-humorous perspective that could strengthen the capacity for reperceiving by inviting a sense of lightheartedness into the present moment (e.g., during meditation, whenever one realizes one exerts too much effort or strives for specific outcomes). In addition, mindful interpersonal humor is a possible way of sharing a moment of lightheartedness with others in daily life.

### 1.2 Humor

Humor in its broadest meaning in daily life and contemporary humor research is used as an umbrella term for everything that is funny, comprising benevolent as well as malicious forms (Martin and Ford, 2018). While having a sense of humor is typically positively connotated in lay usage, in psychological research it is used as a neutral personality characteristic referring to individual differences in (a) humor perception and appreciation, (b) how and beyond which threshold individuals use or indulge in humor, and (c) humor production (Martin and Ford, 2018). However, merely having a sense of humor does not offer any clarification about the kind of humor style a person habitually uses. This is important, because different forms of humor are differentially related to outcomes, such as well-being and mindfulness (Özyeşil et al., 2013; Martin and Ford, 2018). Further, playfulness is considered an essential building block for humor, that can also be described as a play with ideas, and benevolent forms of humor in particular (McGhee, 1999). The ability to take things seriously while maintaining a playful attitude is considered an essential quality for health in adulthood (Winnicott, 1971; McGhee, 2010).

One important taxonomy of humor styles are the *comic styles* (Schmidt-Hidding, 1963; Ruch et al., 2018a), because they distinguish between eight “elementary flavors” of humor: benevolent humor, fun, wit, and nonsense on the lighter side, and cynicism, sarcasm, irony, and satire as darker, or mocking styles. Importantly, in this classification, benevolent humor is exclusively positively defined in contrast to using humor as an umbrella term. Since humor research has taken into account that different forms or styles of humor might be differentially related to well-being, a clearer picture regarding humor and well-being has emerged (for an overview, see Martin and Ford, 2018). Benevolent humor is a unique predictor of positive and negative affect and life satisfaction, explaining subjective well-being beyond age, gender, and personality (Ruch et al., 2018b). It might also be an important correlate of emotion regulation as humorous reappraisals can induce a shift in perspective by reappraising aversive situations or stimuli in a positive or less threatening way facilitating greater emotional distance and reducing negative affect (Samson and Gross, 2012). In contrast, sarcasm and cynicism correlate positively with negative affect, and cynicism correlates negatively with positive affect and life satisfaction (Ruch et al., 2018b). Darker forms of humor may also lead to gelotophobia, the fear of being laughed at (Ruch and Proyer, 2008).

Research into the only standardized humor training for groups, the 7 Humor Habits Program (McGhee, 1999, 2010) has shown that components of the sense of humor can be trained (Ruch and McGhee, 2014) and that the training leads to increases in facets of psychological well-being (self-efficacy, positive affect, and optimism) as well as reductions in perceived stress (Crawford and Caltabiano, 2011). However, existing humor training programs are not sensitive to the varying relationships between different humor styles and well-being. As such, they may mix the training of lighter and darker styles (for an overview, see Ruch and Hofmann, 2017).

### 1.3 Mindfulness humor relationship: intention and attitude

Metzner (2013) suggested that, among other qualities, mindfulness and benevolent humor are inherently based on loving-kindness and benevolence. Still, the relationship was not delineated systematically, and separate exercises were proposed to foster either mindfulness or humor. Later, Hofmann et al. (2019) broadly classified humor into virtuous and non-virtuous forms, suggesting that mindfulness was only linked to virtuous forms of humor by a “core of benevolence” and basal cognitive mechanisms. However, categorizing *entire* humor styles as either virtuous or not imposes two problems. First, focusing only on so-called virtuous forms of humor ignores the fact that all humor styles, even darker ones like irony, can be virtuous, albeit to a lesser extent than benevolent humor (Beermann and Ruch, 2009a). Second, most humor can be considered neutral regarding vice and virtue (Beermann and Ruch, 2009b). Making this strict distinction risks neglecting possible links between other humor styles and mindfulness. To understand which forms of humor might be in alignment with mindfulness, a more fine-grained distinction not only between, but *within* humor styles is warranted.

Such a distinction is made possible by focusing on two essential components of mindfulness and humor: intention and attitude (Schmidt-Hidding, 1963; Shapiro et al., 2006). If mindfulness were misunderstood as mere “non-judgmental awareness” (for clarifications see Dreyfus, 2011), focusing on the attention component only, all forms of humor, including sarcasm or cynicism, would *always* be in alignment with mindfulness. However, mindfulness and humor meet at the tip of intention. Intentions are not only crucial for mindfulness practice (Kabat-Zinn, 2011; Shapiro and Carlson, 2017), but also shape the direction of one's humor, such as whether it is malicious or benign (Schmidt-Hidding, 1963). Only forms of humor that share the underlying intention and attitudes with mindfulness are compatible. The intention should be non-harming (Kabat-Zinn, 2011; Shapiro and Carlson, 2017) and in interpersonal situations always consider others and the context as well, such as whether a situation is appropriate for humor. Forms of humor in line with such an intention are those used to gain insight into the nature of life and experiences, sustain one's spirit in the face of adversity, and perhaps even support one's path to wisdom, compassion, and understanding through ongoing mindfulness practice, which might eventually also serve others (Kabat-Zinn, 2011; Shapiro and Carlson, 2017).

Schmidt-Hidding (1963) outlined the intentions and attitudes underlying each of the eight comic styles (see Ruch, 2012, p. 72, for an overview). Benevolent humor has the intention of arousing “sympathy and an understanding for the incongruities of life, the imperfections of the world, the shortcomings of fellow humans, and the own mishaps and blunders” (Ruch et al., 2018b, p. 3). It is based on a loving, tolerant, and accepting attitude toward oneself and others. Like mindfulness, it can be seen as a quality of the heart. In contrast, sarcasm intends to hurt others, and is grounded in a hostile, derisive, and malicious attitude similar to cynicism, which intends to emit poison and to devaluate generally accepted values. Thus, both being mostly mutually exclusive with mindfulness. The other comic styles may or may not be in alignment with mindfulness depending on the intention and attitude of the individual employing them. For example, irony, as outlined by Schmidt-Hidding (1963), is often based on a critical attitude with the intention to feel superior to others or laugh at them (e.g., framing another person's weaknesses as strengths). This would not be in alignment with mindfulness. But loving-kind self-irony, for example gently laughing about oneself if taking oneself too seriously, might be. So, the consideration of intention and attitude allows for a more fine-grained distinction within comic styles, also including aspects of neutral and darker forms.

### 1.4 Mindful-humorous perspective

This article proposes that forms of humor that share intention and attitude with mindfulness can provide the foundation for a *mindful-humorous perspective*, meaning an internal, humorous outlook on life (e.g., Martin, 2003), and *mindful humor*, meaning a mindful approach to interpersonal humor, which are assumed to rely to varying degrees on different forms of humor. A mindful-humorous perspective fully encompasses benevolent humor, as both are qualities of the heart that intend to arouse insight, well-being, and compassion for the imperfections in the world as well as for one's suffering and that of others (Schmidt-Hidding, 1963; Shapiro and Carlson, 2017). To a lesser degree, it also includes nonsense, which can be considered non-harmful by nature (e.g., to smile about the sheer absurdity of life, and that nothing earthly is constant, even if one tries to cling to it) or neutral and benevolent aspects of fun and irony.

A mindful-humorous perspective is based on a deep and profound inner understanding of the imperfections of life and the human condition, reflecting an insightful knowledge of the ever-changing, inevitable, and often uncontrollable pleasant and unpleasant character of present-moment experiences. This perspective emerges when the open, interested, accepting, and friendly attitude underlying mindful awareness is interfused with a playful frame of mind, inviting a sense of lightheartedness into the present moment. Indeed, Shapiro and Carlson (2017) consider playfulness as an attitudinal foundation of mindfulness, describing it as “bringing a quality of joy, levity, and warmth to one's attention” (p. 18).

A mindful-humorous perspective could support one's mindfulness practice and the cultivation of insights. This can be exemplified through different responses to recurring thoughts during meditation that could be perceived as annoying, intriguing, funny, etc. Such thoughts can be perceived as hindrances to meditation and met with automatic negative evaluations, either because the content of the thoughts is negative or the mind-wandering itself is seen as a problem. This may lead to increased attempts to suppress thinking altogether, undermining the potentially intended outcome (Shapiro et al., 2018). Instead, waves of thoughts can be surfed on, thus, maybe even welcoming them. Seeing the lighter side of being carried away by waves of thoughts can invite a sense of lightheartedness into such moments, potentially supporting a fundamental instruction in mindfulness meditation to fully recognize the process of mind-wandering and to redirect attention gently but firmly to the present moment, seeing thoughts as they are (Kabat-Zinn, 1990). A mindful-humorous perspective can emerge without words or images as mere “internal smile,” for instance, reflecting the deep knowing that one was lost *again*, judging the judging, or clinging to some outcome, and as soon as realizing it, seeing the humor in it, and letting go of it. In addition, it may contain a positive humorous reappraisal (Perchtold et al., 2019), a form of positive reappraisal (Garland et al., 2009), by either seeing a general positive aspect of the experience or finding a positive consequence or side effect of it.

A mindful-humorous perspective is a complementary way of developing a new relationship to experience. Nurturing this perspective may reinforce potentially shared mechanisms and attitudinal foundations between mindfulness and humor. Once intention and attitude are in alignment, both mindfulness and humor support the ability to take the literal “step back” by distancing oneself from a stimulus, facilitating a shift in perspective and dis-identification with one's thoughts, sensations, and emotions (Shapiro et al., 2006; Samson and Gross, 2012). As soon as one spots the humor in *it*, one is no longer it. In this way, a mindful-humorous perspective may also strengthen attitudinal foundations of mindfulness (Shapiro et al., 2006), by making it easier to open up toward the present-moment experience, to perceive it fully and maybe even accept it, just as it is, to investigate its true nature with friendly interest.

While some forms of humor serve as a mere distraction from experience (Samson and Gross, 2012), a mindful-humorous perspective does not involve looking away or denying unpleasant experiences. Rather, it encourages one to look directly at what is painful (like “mature humor,” e.g., Vaillant, 2000), facilitating exposure (Shapiro et al., 2006). A mindful-humorous perspective may therefore make it easier to experience the relativity and transitory nature of thoughts and experiences (Shapiro et al., 2006), and with time, to become more independent from whether current experience is pleasant or unpleasant. Eventually, one might even recognize a mindful-humorous perspective in how attached we are to certain things or try to force certain goals and outcomes. With humor, one can begin to see through the tendencies to strive for pleasant experiences and to avoid unpleasant ones, and maybe let go.

A mindful-humorous perspective is intended to complement commonly used techniques supporting mindfulness meditation (e.g., counting breaths, labeling experiences, or metaphorical instructions “seeing thoughts as clouds in the sky, that come and go”). It should, therefore, always stay in the background, not distract, and neither add to nor detract from the experience, only assist in seeing things as they are. A mindful-humorous perspective may only use a second of gentle remembering and maybe even result in a hint of a smile, returning to the present moment experience, perceiving life as it is, but maybe a little lighter than before. In summary, a mindful-humorous perspective may initiate a positive, self-sustaining feedback-loop, supporting a long-term mindfulness practice and the cultivation of insight and growth.

### 1.5 Mindful humor

A mindful-humorous perspective extends beyond meditation. Mindfulness may facilitate spotting the humor amid the incongruities of daily life in general. In addition, in interpersonal situations, mindful humor may foster positive emotions and relationships by emphasizing shared, inclusive laughter over mockery. This might be encouraged by adopting a playful inner attitude (Winnicott, 1971; McGhee, 2010). Mindful humor has benevolent humor at its foundation but may also include more benevolent or neutral aspects of fun, and to a lesser degree wit, when these are grounded in a playful attitude intended to spread good mood, for example when cheering up a friend. However, fun and wit, that cross the threshold between friendly teasing to mockery (i.e., deriving pleasure from laughing at the expense of others) or wit, that is solely based on the intention to boost one's own ego are not in alignment with mindfulness. To a small extent, mindful humor might also encompass aspects of darker styles that arise from a relaxed and friendly attitude, such as when gently criticizing a friend to correct a behavior or trying to show someone the error of their ways while not feeling superior, but genuinely wanting their benefit with irony. Although satire, sarcasm, and cynicism are in most cases mutually exclusive with mindfulness, satire with a virtuous intention, like aiming to change the societal status quo for the better, might be in accordance with mindfulness, as might rare cases of sarcasm or cynicism if used as the best or only coping mechanism available, such as mocking a toxic work environment in a sarcastic way or reports from survivors of concentration camps (Frankl, 1984).

That said, there is of course no one-size-fits-all approach to interpersonal humor, as even mindful humor based on the best intentions could be misread as mockery or as being inappropriate for the situation. It is the responsibility of each individual to mindfully observe one's intentions, the situation, and the consequences humor has on others, to decide whether mindful humor would be a skillful means. Ideally, mindful humor should have positive effects, or at least no detrimental effects, for the individual and others, thus potentially initiating a second positive, self-sustaining feedback loop.

### 1.6 Mindful humor filter model

**Figure 1:**
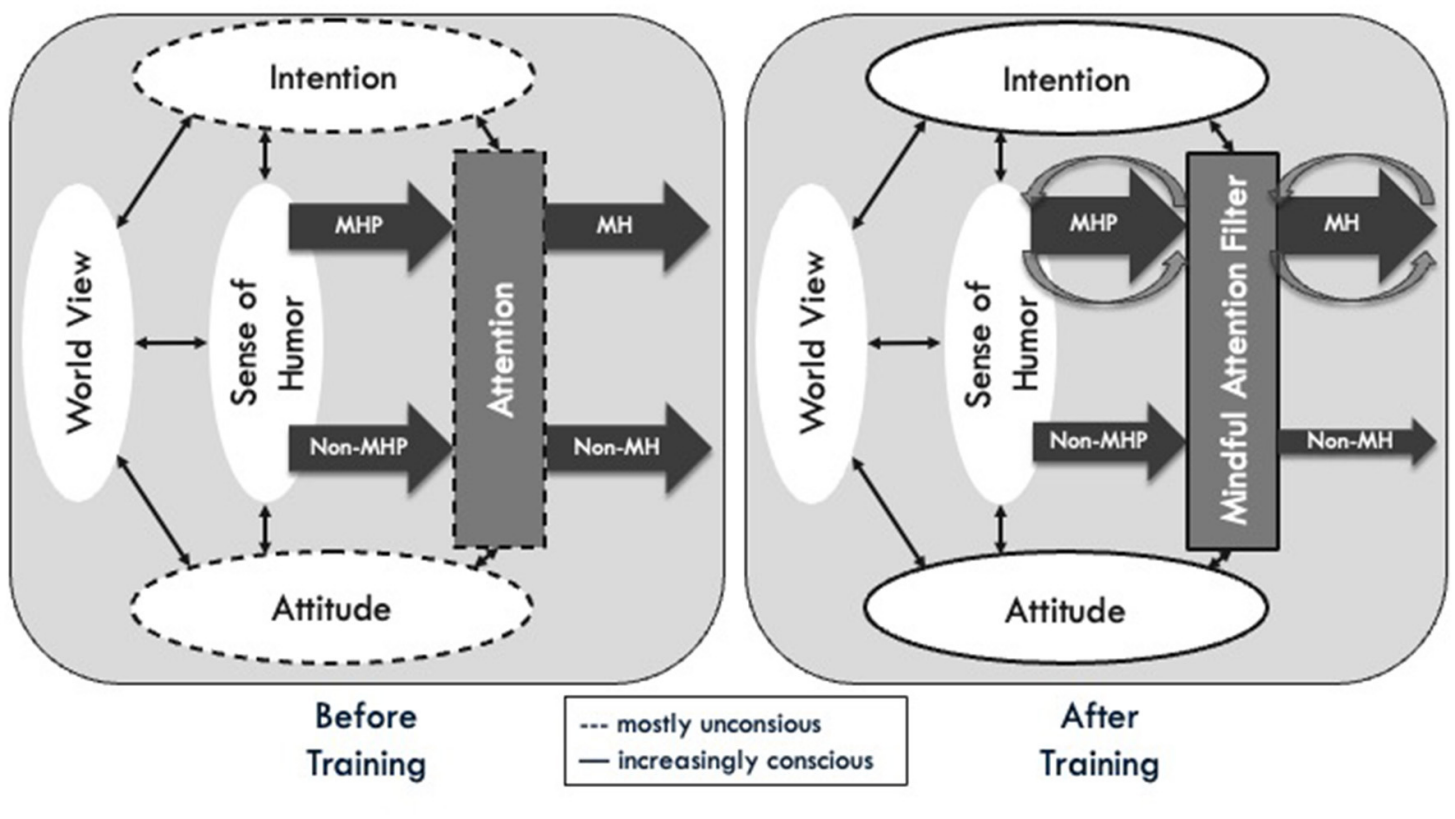
The mindful humor filter model (MHFM) before **(left)** and after **(right)** training. Mindfulness and humor are interconnected via intention, attention, and attitude, which are initially assumed to be mostly unconscious, thus depicted as dotted lines **(left)**. With increasing levels of mindfulness **(right)**, they become more and more conscious (solid lines), leading to an increase in the habitual occurrence of a mindful-humorous perspective (MHP) and mindful humor (MH) and a decrease in non-MHP and non-MH (e.g., sarcasm or cynicism). The decrease is assumed to be smaller for non-MHP, because the change in one's habitual humor perspective is assumed to happen in the long-term, while the inhibition of non-MH may be easier achieved. MHP and MH both go along with a two-component positive reinforcement loop. The first is internal and refers to reinforcing shared mechanisms and attitudinal foundations through a MHP. The second is external and refers to MH fostering positive emotions and relationships.

A mindful-humorous perspective and mindful humor can occur naturally but can also be cultivated by explicitly bringing intention and attitude to one's awareness. The mindful humor filter model summarizes the hypothesized mindfulness-humor relationship before and after training (Figure 1). It further describes the assumed changes that occur during a mindfulness-based program, including the two postulated feedback loops initiated by a mindful-humorous perspective and mindful humor. In the model, the intention, attention, and attitude of one's habitual use of humor are initially assumed to be mostly implicit and unconscious (Martin et al., 2003). The habitual humor of an individual is determined by its sense of humor that is mostly shaped by past experiences and conditioning (McGhee, 2010), including a general world view (or primal world beliefs, Clifton et al., 2019) that determines the individual's perspective on a stimulus or situation and its subsequent interpretation.

As mindfulness and humor are linked via intention, attention, and attitude, with ongoing mindfulness practice, the underlying intention and attitudinal foundations should be continuously strengthened, initiating a change toward increased habitual occurrence of a mindful-humorous perspective and mindful humor and less other forms of humor. In addition, mindfulness may be helpful in spotting incongruities and inviting a mindful-humorous perspective, particularly in situations where humor might not be immediately evident. This may be facilitated through reperceiving and broadened attention (Garland et al., 2009), until with practice, a mindful-humorous perspective might occur more often habitually. Further, mindfulness does provide a space (Kabat-Zinn, 1990), to decide within the present moment, whether a situation is appropriate for humor (mindful humor as well as other forms), or to reflect on whether one wants to make a potentially hurtful humorous comment. In this way, the present-centered, accepting, and friendly awareness of mindfulness may foster a mindful-humorous perspective and mindful humor while at the same time filtering out the darker aspects of lighter styles (e.g., hurtful aspects of fun), as well as inhibiting or reducing destructive forms of humor (e.g., sarcasm, cynicism) or maybe even transform them into a mindful-humorous perspective and mindful humor.

The model includes two postulated feedback loops (see Figure 1). First, a mindful-humorous perspective may initiate a self-sustaining momentum, therefore, increase the likelihood of starting and sustaining one's own mindfulness practice, which can facilitate the cultivation of insights and wisdom. Further, mindfulness practice might facilitate a shift in perspective, toward a mindful-humorous outlook on life in general, thus maybe even transforming one's world view in the long term. Second, mindful humor can induce positive emotions in the self and others, being a “social lubricant” that helps foster positive relationships.

### 1.7 Existing research on mindfulness and humor

Existing empirical research on the relationship between mindfulness and humor is almost exclusively atheoretical and based on cross-sectional studies that indicate a positive relationship between mindfulness (or facets of mindfulness) and adaptive (affiliative and self-enhancing, coping humor) or light forms (benevolent humor, wit) of humor, and a negative relationship with maladaptive (aggressive, self-defeating) or darker forms (sarcasm, cynicism) of humor (Özyeşil et al., 2013; Khramtsova and Chuykova, 2016; Geiger et al., 2019; Hofmann et al., 2019; Pérez-Aranda et al., 2021; Saricali et al., 2022). The only longitudinal study to date found that participants of MBSR and mindfulness-based strengths practice (Niemiec, 2014) showed a significant increase in a virtuous form of humor (i.e., humor as a character strength; Peterson and Seligman, 2004) after training, which was sustained up to 6 months (Hofmann et al., 2019). However, there is no existing program, which is sensitive to the distinction between lighter and darker forms of humor, or that specifically aims to foster benevolent humor (Ruch and Hofmann, 2017). Due to the assumed synergetic effects between mindfulness and forms of humor that share intention and attitude with mindfulness, a joint training might be especially fruitful in encouraging mindfulness practice and improving well-being (Özyeşil et al., 2013; Khramtsova and Chuykova, 2016; Hofmann et al., 2019).

### 1.8 Aims and hypotheses

The objectives of the present study are as follows: firstly, it aims to develop a humor-enriched mindfulness-based program (HEMBP) based on the theoretical framework of the mindful humor filter model (Figure 1) with the explicit goal of nurturing a mindful-humorous perspective and encouraging mindful humor. Secondly, the study seeks to rigorously assess the validity and efficacy of the HEMBP as a first test of the integration of mindfulness and humor using a randomized controlled trial. The research will provide valuable insights into the program's effectiveness in enhancing well-being, reducing stress, and fostering a mindful-humorous approach to life. The randomized trial serves as the first step in a series of studies to assess the mindful humor filter model more comprehensively.

In line with the mindful humor filter model, it is hypothesized that participation in the HEMBP is associated with increases in mindfulness, benevolent humor, psychological well-being, and life satisfaction, and decreases in sarcasm, cynicism, stress, and gelotophobia, from pre-test to post-test and from pre-test to 1-month follow-up, compared to individuals in a wait-list control group. No changes are predicted for the other comic styles as they are not the focus of the training (i.e., nonsense) and encompass potentially hurtful and non-hurtful elements (i.e., fun, wit, irony, satire).

## 2 Methods

### 2.1 Participants

Eligible participants were adults aged 18 years or over with sufficient command of German to understand instructions. Exclusion criteria were previous experience in mindfulness meditation and currently being in psychotherapeutic treatment. The final sample consisted of 60 participants (65% female, 35% male) aged between 18 and 63 years (*M* = 36.02, *SD* = 11.91) who were matched for age and gender and randomly assigned to one of two conditions: the HEMBP intervention group (*n* = 30), or a wait-list control group (WL; *n* = 30). Participants' demographic data are displayed in Table 1.

**Table 1:** Demographic data of participants and results of between-group comparisons at pre-test and for completion status.

**Variable**	**Pre-test**				**Completion**	
	**HEMBP (*****n*** = **30)**	**WL (*****n*** = **30)**	χ^2^ **(*****df*****) (*****N*** = **60)**	***p***	χ^2^ **(*****df*****) (*****N*** = **60)**	***p***
Gender, *n* (%)			0.07 (1)	0.787	2.66 (1)	0.103
Male	10 (33.3%)	11 (36.7%)				
Female	20 (66.7%)	19 (63.3%)				
Highest level of education, *n* (%)			2.93 (5)	0.710	7.63 (5)	0.178
Secondary school	1 (3.3%)	0 (0.0%)				
Apprenticeship	4 (13.3%)	5 (16.7%)				
Baccalaureate school	5 (16.7%)	3 (10.0%)				
Bachelor's degree	13 (43.3%)	14 (46.7%)				
Master's degree	6 (20.0%)	8 (26.7%)				
Doctorate	1 (3.3%)	0 (0.0%)				
Nationality, *n* (%)			0.38 (3)	0.945	1.94 (3)	0.585
Swiss	23 (76.7%)	21 (70.0%)				
German	3 (10.0%)	4 (13.3%)				
Austrian	1 (3.3%)	1 (3.3%)				
Other	3 (10.0%)	4 (13.3%)				
Employment status ^a^, *n* (%)						
Working full-time	14 (46.7%)	10 (33.3%)	1.11 (1)	0.292	0.09 (1)	0.768
Working part-time	8 (26.7%)	15 (50.0%)	3.46 (1)	0.063	0.17 (1)	0.683
Studying full-time	1 (3.3%)	5 (16.7%)	2.96 (1)	0.085	1.18 (1)	0.278
Studying part-time	3 (10.0%)	2 (6.7%)	0.22 (1)	0.640	0.11 (1)	0.744
Housekeeping	2 (6.7%)	3 (10.0%)	0.22 (1)	0.640	0.11 (1)	0.744
Retirement	1 (3.3%)	0 (0.0%)	1.02 (1)	0.313	0.18 (1)	0.672
Unemployment	4 (13.3%)	1 (3.3%)	1.96 (1)	0.161	0.96 (1)	0.327
			*t* (*df*)	*p*	*t* (*df*)	*p*
Age, *M* (*SD*)	35.2 (11.2)	36.8 (12.7)	0.51 (58)	0.615	−0.39 (58)	0.700

HEMBP, humor-enriched mindfulness-based program; WL, wait-list control group; Pre-test, Comparison between conditions (HEMBP vs. wait-list control group) at pre-test; Completion, Comparison between completers (*n* = 52) and non-completers (*n* = 8). ^a^For employment status, multiple responses were possible. Thus, the *n* and percentage in each cell represent those that selected the respective option (out of 30 each).

**Figure 2:**
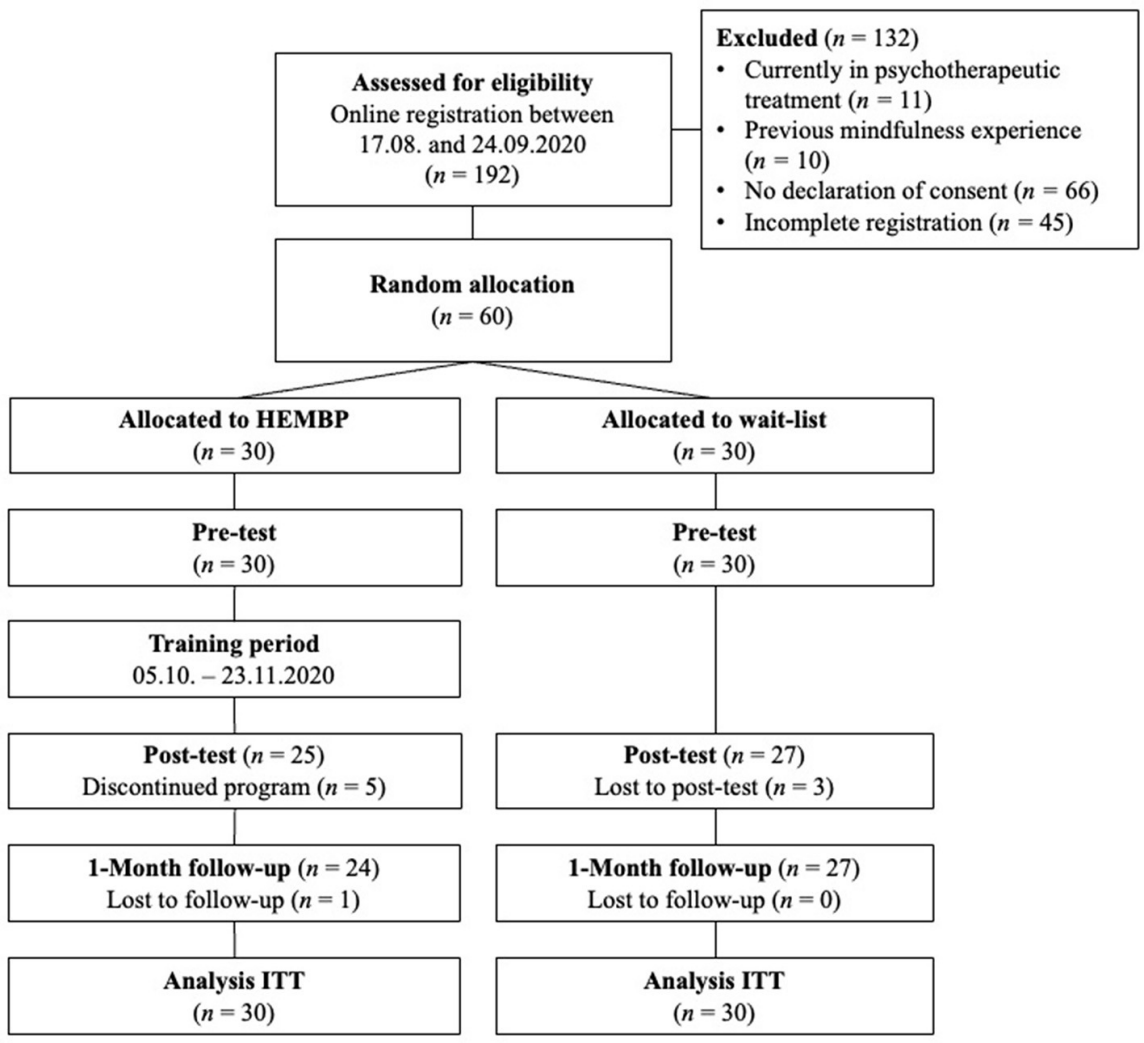
Participants flowchart in accordance with CONSORT criteria. HEMBP, humor-enriched mindfulness-based program; ITT, intention-to-treat.

Most participants were Swiss (73%), German (12%), or Austrian (3%). More than two-thirds of the participants (70%) had a bachelor's degree or higher. The majority of participants was employed either full- (*n* = 23) or part-time (*n* = 24), while 11 participants were full- or part-time students, five were housewives/husbands, one was retired, and five were unemployed. As shown in the participant flow chart (Figure 2), 60 participants completed the pre-test, 52 the post-test, and 51 the 1-month follow-up test, giving an 85% retention rate. Participants indicated the following reasons for discontinuing the program: lack of time (*n* = 3), no added value (*n* = 1), and psychological problems unrelated to the program (*n* = 1). Due to the COVID-19 pandemic situation, one person decided after three sessions to not continue the program in person but continued to practice based on audio recordings and course materials and completed all assessments. This participant's data was included in all analysis as this did not change results.

### 2.2 Procedure

Participants were recruited between August 17^th^ and September 24^th^, 2020 via flyers, social networks, the project home page, and direct mailing to companies, all of which included a link to the web-based survey. The training was advertised as a mindfulness-based program that integrated the latest research on mindfulness and humor to foster a mindful-humorous outlook on life and mindful humor that encouraged sympathy and understanding for the imperfections of life. During the online registration process, participants who met inclusion criteria were informed about the study and asked to give written consent, before providing basic demographic data. Personalized feedback based on participants' answers to the self-report measures was offered as an incentive. Participants were required to pay CHF 100 for participation to motivate them and to reduce drop-out. The Ethics Committee of the Faculty of Arts and Social Sciences of the University of Zurich reviewed and approved the study (reference number: 20.8.2).

After matching participants by age and gender, they were randomly assigned to one of the two conditions using a computer-generated randomization list, concealed to the author until all participants were randomized. Participants were asked to complete the same self-reports online on three occasions: before the start of the program, after its completion, and at a 1-month follow-up. The training was implemented as a face-to-face group program split into two sub-groups (*n* = 15 each) to ensure the quality of program delivery. The HEMBP follows a standardized 8-week manual. It was delivered by the author, a qualified MBSR teacher (acknowledged by the Swiss Mindfulness Association), between October 5^th^ and November 23^rd^, 2020, in a classroom at the University of Zurich. Participants met on eight consecutive Mondays for 2.5-h sessions. Written course materials and guided audio mindfulness meditations were provided. The trainings started at very low COVID-19 rates. Due to rapidly increasing COVID-19 rates 2 weeks after the start of the training, online participation via Zoom was enabled and the all-day mindfulness session was canceled. Ten participants chose to participate online for 3.13 classes on average. The wait-list control group received the same training after data collection had been completed.

### 2.3 Humor-enriched mindfulness-based program design

#### 2.3.1 Structure and content

The HEMBP was designed based on the mindful humor filter model, by restructuring MBSR (Kabat-Zinn, 1990), to incrementally incorporate (adapted) humorous elements from the 7 Humor Habits Program (McGhee, 2010) and newly designed, integrated exercises for mindfulness and humor. It consists of eight 2.5-h weekly group sessions and a 6.5-h day session. The program is led by a qualified HEMBP teacher. Participants learn to systematically engage in formal mindfulness practices such as body scan, yoga, and sitting-, loving-kindness-, walking-, and tree meditation, and in informal practices whereby mindfulness is integrated into daily activities. Recommended homework (≥30-min/day) consists of formal (≥20-min/day, 6 days/week) and informal mindfulness practice, exercises, and calendars. As the HEMBP was initially intended for healthy participants, the duration of guided meditations was abbreviated to reduce the threshold to begin and maintain a regular, long-term mindfulness practice. Further, there is as yet no consistent evidence for a positive relationship between formal practice time and outcomes (Parsons et al., 2017; Lloyd et al., 2018). The weekly in-person sessions include (a) opening and closing meditations, (b) a summary of the last session and outlook on the current one, (c) mindfulness practices and discussion in small and/or large groups, (d) introduction of new mindfulness meditation and/or theme, (e) experiential-based exercises and games, and (f) homework.

Table 2 gives an overview of the contents and exercises of the HEMBP. The HEMBP is built around two core themes. First, the program structure is designed to best support participants in starting and sustaining a long-term mindfulness practice through a mindful-humorous approach. Second, weekly gatherings are intended to provide a safe learning space in which the acquired mindfulness skills can be applied to develop a new relationship to experience to foster well-being and to reduce stress through the cultivation of insights and understanding. The focus of the HEMBP is on fostering positive qualities (e.g., mindful–humorous perspective, mindful humor, loving-kindness, compassion, gratitude, equanimity, and positive relationships) in alignment with positive psychological interventions (Sin and Lyubomirsky, 2009), though it also includes one class on stress reduction, as both pathways to well-being should be addressed in a complete MBP.

**Table 2:** Course overview: humor-enriched mindfulness-based program (HEMBP).

**Class**	**Title and theme**	**Mindfulness practices and exercises**	**Home practice**
1	Arrival–Mindfulness–Autopilot: Mindful awareness allows one to give full attention to the present moment as life is unfolding exclusively in the here and now, as well as learning, growth, and transformation.	Tree meditation; Body scan Raisin-eating exercise	Formal: Body scan, tree meditation; Informal: One breath, routine activity General: Tips for habit formation
2	Lightheartedness: Aimlessness (mindful-playful perspective) and loving- kindness as central attitudinal foundations of mindfulness and a new way of relating to experience (regardless of whether pleasant, unpleasant, or neutral).	Body scan; Introduction to sitting meditation (mindfulness of breathing) and loving-kindness meditation Aimless vs. competitive (goal) ball game; Paper tiger	Formal: Body scan; Formal / Informal: Loving-kindness meditation / mindfulness of breathing (separately or integrated), 9 dots puzzle Mindful-playful lightheartedness: Informal (mindful-playful routine); Choosing mindful-playful reminders; Moments of lightheartedness in daily life: Paper tiger and reflection
3	How we perceive the world: Perception and interpretation of internal and external stimuli shape decisively how they affect body and mind. Introduction of a humor perspective.	Sitting meditation; Yoga (lying down); Short, guided meditation with a mindful-humorous perspective Review of 9-dots puzzle	Formal: Body scan or mindful yoga in alternation; Formal / informal: loving-kindness meditation / mindfulness of breathing Humor: Surround yourself with humor; Calendar (self-observation) of humor experiences, accompanying thoughts, bodily sensations, and feelings, as well as one's associated intention and humor perspective
4	A mindful-humorous perspective (MHP): Life is not perfect. A mindful-humorous perspective allows seeing the brighter side of things and to invite a sense of lightheartedness into the present moment regardless of the circumstances.	Sitting meditation; Standing yoga Review of humor experiences calendar Exercise for MHP during meditation	Formal: Body scan or mindful yoga in alternation; Formal / Informal: Loving-kindness meditation / mindfulness of breathing Mindful-humorous: Calendar for humor experiences (focus on MHP) in daily life and accompanying thoughts, bodily sensations, and feelings, as well as one's associated intention and humor perspective
5	Mindful humor in everyday life: Mindful humor as a means to share a moment of lightheartedness with others, fostering positive, mindful interpersonal relationships.	Sitting meditation; Standing yoga Midway reflection; Review of calendar of MHP in daily life; Exercise for MHP and mindful humor in everyday life	Formal: Mindful yoga or sitting meditation in alternation; Formal / Informal: Loving-kindness meditation Mindful-humorous: Exercises for MHP and mindful humor in daily life; Calendar of pleasant (if possible: humorous) experiences
6	Savoring and gratitude: Mindfully exploring common features of pleasant experiences and discovering them as a possibility of savoring while practicing letting go of them. Cultivating gratitude for neutral experiences.	Sitting meditation; Yoga (lying down) Review of calendar of pleasant (humorous) experiences and of exercises for a MHP and mindful humor in daily life Chocolate meditation; Gratitude meditation	Formal: Body scan, mindful yoga, or sitting meditation in alternation; Formal / Informal: Loving-kindness meditation Mindful-humorous: Calendar of unpleasant (if possible: humorous) experiences; Savoring and letting go (pleasant experiences); Gratitude (neutral experiences)
7	Mindful coping with stress: Mindfulness offers choices in stressful situations and when facing unpleasant experiences in order to respond positively and proactively instead of habitual automatic reactions.	Sitting meditation; Standing yoga Review of calendar of unpleasant (humorous) experiences Worksheet (exploration of individual stress reactivity cycle)	Free choice
	Day of Mindfulness: A practice day for the cultivation of seamless continuity of mindful awareness between formal as well as informal mindfulness practices.	Formal practice: Body scan, mindful yoga, sitting meditation, walking meditation, tree meditation Informal practice: Mindful eating Mindful-playful ball game “balls vs. food”	
8	Rest of your life: The end of this journey is the start of an ongoing integration of mindfulness and a mindful-humorous perspective into one's daily life, maintaining a continuous, long-term mindfulness practice.	Practice sequence: Body scan, lying down yoga, sitting meditation Review of stress reactivity cycle and mindful responding	Deepening of mindfulness practice and review of the program Tips for reinforcing and sustaining an ongoing mindfulness practice Recognizing and cultivating “progress” in one's own practice

MHP, Mindful-humorous perspective.

In alignment with the mindful humor filter model, the program first focuses on the cultivation of mindful awareness. Throughout the program, mindful-playful/-and humorous elements are incrementally integrated. In class 2, loving-kindness is introduced together with a mindful-playful exploration of present-moment experience. This strengthens the attitudinal foundations of mindfulness, creating an internal climate of friendliness. This approach lays the foundation for introducing perspectives (class 3) and a mindful-humorous perspective during meditation (e.g., when putting too much effort into one's meditation) and in daily life (e.g., when facing daily mishaps) as a complementary way of forming a new relationship to experiences enabling participants to invite a sense of lightheartedness into the present moment (class 4). Mindful humor is presented as a way of sharing such a moment with others to foster positive emotions and relationships (class 5). The rest of the program focuses on the exploration of pleasant, neutral (class 6), and unpleasant (stress; class 7) experiences while deepening one's mindfulness practice. Experience-based learning is further facilitated through mindful-playful exercises such as ball games to explore the difference between intention and goal-orientation, and calendars to explore the consequences of mindful- and non-mindful forms of humor and whether these are in alignment with one's values.

Emphasis was placed on keeping the “warp” elements of an MBP intact (Crane et al., 2017), namely (a) the theoretical foundations of MBPs remain unchanged, as well as the HEMBP (b) offers guidance on fostering well-being and reducing distress based on a model of human experience, (c) teaches a mindful-humorous perspective as a complementary way of developing a new relationship to present moment experience reinforcing reperceiving and related mechanisms (e.g., self-regulation, approach orientation), as well as core attitudinal foundations (openness, curiosity, acceptance, and friendliness), (d) through a systematic training in mindfulness meditation, and (e) an experiential, insightful inquiry-based learning process (e.g., supporting participants to discover for themselves how perception shapes how they think and feel). The HEMBP curriculum can be accessed by contacting the author.

#### 2.3.2 Development process

Prior to this study, the HEMBP curriculum was developed in two phases. First, the new curriculum was designed and then tested for feasibility and acceptance with a convenience sample. The program was continuously adapted and refined based on feedback from participants through group and individual interviews and evaluation questionnaires (e.g., whether provided material, exercises, and explanations were understandable and acceptable), from experts in mindfulness and humor such as experienced MBSR teachers and researchers and practitioners. New exercises were pre-tested in the author's meditation group and a revised curriculum was tested again before this study with a convenience sample interested in mindfulness meditation but without mindfulness experience. The curriculum was developed by the author, a certified MBSR teacher (teacher formation 2014/2015, 3 years of teaching experience, 12 years of mindfulness meditation experience, >40 days of vipassana/zen retreat experience prior to this study).

### 2.4 Measures

**Comic Style Markers** (CSM; Ruch et al., 2018a). The CSM is a 48-item questionnaire measuring eight comic styles: fun, benevolent humor, nonsense, wit, irony, satire, sarcasm, and cynicism. An example item for benevolent humor is “on a large and small scale, the world is not perfect, but with a humorous outlook on the world I can amuse myself at the adversities of life.” There are six items for each scale, utilizing a 7-point response format from 1 (*strongly disagree*) to 7 (*strongly agree*). In the current study, the internal reliability across measurement occasions ranged from α = 0.73 (irony) to 0.94 (nonsense).

**Comprehensive Inventory of Mindfulness Experiences** (CHIME; Bergomi et al., 2014). The CHIME provides a multi-dimensional assessment of mindfulness on eight subscales: awareness of internal and external experiences, acting with awareness, openness to experiences, accepting and non-judgmental orientation, decentering and non-reactivity, insightful understanding, and relativity of thoughts. A reverse coded example item for openness to experiences is “when I am in pain, I try to avoid the sensations as much as possible.” Answers are given on a 6-point scale from 1 (*almost never*) to 6 (*almost always*). In this study, internal reliability across measurement occasions ranged from α = 0.85 to 0.92.

**Comprehensive Inventory of Thriving** (CIT; Su et al., 2014). The CIT assesses subjective as well as psychological well-being (positive functioning) across 18 subscales that cover seven well-being dimensions: relationships, engagement, mastery, autonomy, meaning, optimism, and subjective well-being. It comprises 54 items, such as “I have found a satisfactory meaning in life” for meaning, typically rated on a 5-point Likert scale, though this was extended in the current study to a 7-point scale of 1 (*strongly disagree*) to 7 (*strongly agree*) to accommodate for potential ceiling effects. In this study, the German version (Hausler et al., 2017) was employed with internal reliability across measurement occasions ranging from α = 0.95 to 0.97.

**GELOPH<15>** (Ruch and Proyer, 2008). The Geloph is a self-report questionnaire to assess gelotophobia, the fear of being laughed at. It consists of 15 items, such as “when strangers laugh in my presence I often relate this to me personally,” measured on a 4-point Likert scale from 1 (*strongly disagree*) to 4 (*strongly agree*). Those not afraid of being laughed at fall at the lower end of the scale, while those at the higher end may have a pathological level of symptoms. Recommended cut-offs are 2.5–3.0 for slight expression of gelotophobia and 3.0–4.0 for pronounced expression. In this study, internal reliability ranged between α = 0.90 and 0.93 across measurement occasions.

**Perceived Stress Scale-10** (PSS-10; Cohen et al., 1983). The PSS measures perceived stress during the past month. The scale consists of 10 items, such as “In the last month, how often have you found that you could not cope with all the things that you had to do?,” with a 5-point Likert scale from 1 (*never*) to 5 (*very often*). The 10-item German version of the PSS was used (Klein et al., 2016), with internal reliability ranging from α = 0.84 to 0.87 across measurement occasions.

**Satisfaction with Life Scale** (SWLS; Diener et al., 1985). The SWLS is a 5-item questionnaire that measures personal evaluation of satisfaction with life in general, such as “in most ways my life is close to my ideal.” It uses a 7-point scale ranging from 1 (*strongly agree*) to 7 (*strongly disagree*). In this study, the German version (Ruch et al., 2010) of the scale was used. Internal reliability was between α = 0.88 and 0.91 across measurement occasions.

All analyses is based on the respective mean total score of the above cited measures.

**Practice time**. At post- and follow-up tests, participants were asked how long they typically practiced formal meditation during the training period and since post-test, measured in average minutes per day.

### 2.5 Statistical analysis

The sample size was estimated via a priori power analyses with G^*^Power 3.1 (Erdfelder et al., 2009). Prior research (Sedlmeier et al., 2017) indicated the global effect size for meditation studies for healthy participants was $\bar{r}\;=\;0.2$7. With an assumed α = 0.05, power = 0.95, and an expected correlation of 0.50 among repeated measures, the required sample size was at least *N* = 38 for a repeated-measures design testing a within-between interaction.

Due to the hierarchical data structure, a set of linear mixed-effects models was applied. The repeated measures (1 week prior to program, 1 week after, and 1-month follow-up; Level-1) are nested within participants (Level 2). Time and condition were entered as dummy coded factors with pre-test and wait-list control group as reference categories. The Level 1 model captures within-person changes in the outcome variables between pre- and post-test, and between pre-test and 1-month follow-up, referred to as slopes (i.e., post-test and 1-month follow-up, respectively). The Level 2 model represents assigned condition (WL, HEMBP). The intervention effect was examined by evaluating the interactions between time and condition, which reflect group differences in the slopes from pre- to post-test, and pre-test to 1-month follow-up. Linear mixed-effects models can estimate model coefficients without list-wise exclusion of cases due to missing data, such that performing an intention-to-treat analysis in accordance with CONSORT guidelines (Schulz et al., 2010) does not require multiple imputation beforehand (Twisk et al., 2013). The “lme” function from the “nlme” package (Pinheiro et al., 2021) in R (R Core Team, 2021) was used for the linear mixed-effects model analyses to set the residual variance to a value close to zero (i.e., 0.0000000001) as zero is not possible. This step is crucial since the model equation at Level 1 only serves to split two measures of each individual into the pre-test score (intercept) and the difference score (slopes), either between pre-/post-test or pre-test/follow-up. Thus, there is no Level 1 residual term (Lischetzke et al., 2015).

To investigate whether formal mindfulness practice outside of the classroom was related to training outcomes, Pearson product-moment correlation coefficients between averaged practice time during training and outcome change scores from pre- to post-test were calculated.

## 3 Results

Differences in demographics and outcome variables between the two groups at pre-test were tested with independent *t-*tests for continuous variables and Chi-square tests for categorical variables. There were no significant between-group differences at pre-test in terms of gender, age, education, nationality, and employment status, as well as for mean levels of mindfulness, fun, humor, nonsense, wit, irony, satire, sarcasm, cynicism, gelotophobia, psychological well-being, life satisfaction, and perceived stress, suggesting the randomization created initially comparable groups. Table 1 shows the test statistics for the group comparisons at pre-test for the demographic variables and Table 3 displays the descriptive data (means and standard deviations) as well as the test statistics for the group comparisons at pre-test for the outcome variables. Additionally, Table 4 presents a correlation matrix for all outcome variables at pre-test for a better understanding of the relationships between those variables.

**Table 3:** Descriptive data for both conditions at three measurement occasions and results of between-group comparisons at pre-test for outcome variables.

**Outcome variable**	**Pre-test**					**Post-test**			**1-Month follow-up**		
	***n***	***M***	***SD***	***t(df)***	***p***	***n***	***M***	***SD***	***n***	***M***	***SD***
Mindfulness				−0.16 (58)	0.872						
WL	30	3.67	0.38			27	3.66	0.49	27	3.68	0.45
HEMBP	30	3.69	0.48			25	3.97	0.51	24	4.07	0.52
Fun				0.11 (58)	0.915						
WL	30	4.34	0.96			27	4.40	0.89	27	4.33	0.92
HEMBP	30	4.32	1.04			25	4.45	1.09	24	4.65	1.06
Humor				−0.75 (58)	0.459						
WL	30	4.88	0.83			27	4.93	0.89	27	4.72	0.87
HEMBP	30	5.05	0.90			25	5.28	0.63	24	5.19	0.87
Nonsense				−0.46 (58)	0.650						
WL	30	4.58	1.25			27	4.49	1.44	27	4.72	1.58
HEMBP	30	4.73	1.20			25	4.83	1.21	24	4.80	1.26
Wit				−0.75 (58)	0.458						
WL	30	4.41	0.91			27	4.57	1.16	27	4.41	1.22
HEMBP	30	4.61	1.15			25	4.60	0.98	24	4.67	0.90
Irony				−0.54 (58)	0.589						
WL	30	4.09	1.03			27	4.24	1.18	27	4.17	1.12
HEMBP	30	4.23	0.95			25	3.87	1.07	24	4.06	1.11
Satire				0.56 (58)	0.574						
WL	30	4.24	1.00			27	4.36	1.10	27	4.26	0.98
HEMBP	30	4.10	0.99			25	4.15	1.01	24	4.01	0.95
Sarcasm				0.68 (58)	0.499						
WL	30	3.65	1.13			27	3.82	1.49	27	3.80	1.42
HEMBP	30	3.45	1.15			25	2.82	1.07	24	2.93	1.06
Cynicism				0.34 (58)	0.732						
WL	30	3.68	1.28			27	3.87	1.51	27	3.86	1.32
HEMBP	30	3.56	1.46			25	3.16	1.27	24	3.13	1.41
Gelotophobia				0.69 (58)	0.490						
WL	30	1.94	0.64			27	1.97	0.60	27	1.97	0.63
HEMBP	30	1.82	0.62			25	1.61	0.47	24	1.54	0.44
PWB				−0.55 (58)	0.586						
WL	30	5.02	0.67			27	4.98	0.73	27	5.03	0.74
HEMBP	30	5.11	0.60			25	5.37	0.76	24	5.55	0.63
Life satisfaction				−0.58 (58)	0.564						
WL	30	4.49	1.09			27	4.43	1.23	27	4.53	1.16
HEMBP	30	4.67	1.31			25	4.93	1.27	24	5.19	1.10
Stress				−1.31 (58)	0.194						
WL	30	2.81	0.38			28	2.89	0.58	27	2.74	0.59
HEMBP	30	3.00	0.68			25	2.64	0.59	24	2.60	0.57

WL, wait-list control; HEMBP, humor-enriched mindfulness-based program; PWB, psychological well-being. Test statistics report whether there was a significant difference in mean outcome levels at pre-test between conditions (HEMBP vs. wait-list control).

**Table 4:** Pearson correlations of the outcome variables at pre-test.

**Outcome variable**	**(1)**	**(2)**	**(3)**	**(4)**	**(5)**	**(6)**	**(7)**	**(8)**	**(9)**	**(10)**	**(11)**	**(12)**	**(13)**
(1) Mindfulness	(0.85)												
(2) Fun	−0.15	(0.79)											
(3) Humor	0.32^*^	0.50^**^	(0.74)										
(4) Nonsense	0.08	0.46^**^	0.58^**^	(0.88)									
(5) Wit	0.02	0.51^**^	0.54^**^	0.42^**^	(0.82)								
(6) Irony	−0.04	0.47^**^	0.46^**^	0.47^**^	0.46^**^	(0.73)							
(7) Satire	0.02	0.44^**^	0.56^**^	0.56^**^	0.44^**^	0.58^**^	(0.77)						
(8) Sarcasm	−0.07	0.28^*^	0.39^**^	0.37^**^	0.51^**^	0.58^**^	0.69^**^	(0.79)					
(9) Cynicism	−0.14	0.29^*^	0.35^**^	0.49^**^	0.42^**^	0.59^**^	0.63^**^	0.69^**^	(0.89)				
(10) Gelotophobia	−0.31^*^	−0.09	−0.11	0.12	−0.18	0.13	0.14	0.18	0.22	(0.92)			
(11) PWB	0.52^**^	0.02	0.24	−0.22	−0.02	−0.16	−0.01	−0.21	−0.25	−0.44^**^	(0.94)		
(12) Life satisfaction	0.42^**^	−0.10	0.12	−0.24	0.03	−0.21	−0.08	−0.20	−0.26^*^	−0.36^**^	0.83^**^	(0.89)	
(13) Stress	−0.53^**^	−0.02	−0.29^*^	−0.01	−0.05	0.26^*^	< 0.01	0.23	0.29^*^	0.31^*^	−0.52^**^	−0.51^**^	(0.84)

PWB, psychological well-being. Internal consistencies (Cronbach's alpha) presented on the diagonal. *N* = 60. ^*^p < 0.05. ^**^p < 0.01.

Potential differences between completers and dropouts were investigated with a series of *t* tests for continuous variables or Chi-square tests for categorical variables. No significant differences by completion status were found at pre-test for any demographic variables (Table 1), or for mean levels of mindfulness [*t*_(58)_ = −1.57, *p* = 0.123], fun [*t*_(58)_ = −0.37, *p* = 0.713], humor [*t*_(58)_ = −1.18, *p* = 0.244], nonsense [*t*_(58)_ = −0.08, *p* = 0.938], wit [*t*_(58)_ = −0.21, *p* = 0.831], irony [*t*_(58)_ = −0.86, *p* = 0.391], satire [*t*_(58)_ = −0.10, *p* = 0.918], sarcasm [*t*_(58)_ = 0.35, *p* = 0.725], cynicism [*t*_(58)_ = 0.38, *p* = 0.707], gelotophobia [*t*_(58)_ = 1.15, *p* = 0.255], psychological well-being [*t*_(58)_ = −0.64, *p* = 0.526], life satisfaction [*t*_(58)_ = −0.77, *p* = 0.446], or perceived stress [*t*_(58)_ = −1.57, *p* = 0.123]. Further, dropout rates did not significantly differ between the two groups, indicating no systematic dropout [$\chi_{\left(1,60\right)}^{2}$ = 1.18, *p* = 0.278].

On average, HEMBP participants attended 6.8 (*SD* = 1.2) sessions, with 84% (*n* = 21) attending ≥6 sessions. Reported average daily formal practice time was 15 min (*SD* = 8.63), corresponding to 75% of the recommended amount of 20 min. A substantial number of participants who completed the follow-up assessment (91.6%) continued engaging in formal mindfulness practice after post-test, with an average of 12.67 min (*SD* = 9.44) per day.

### 3.1 Program effectiveness

Program effectiveness was evaluated by examining differences in slopes of outcome scores between pre- and post-test (post-test), and between pre-test and follow-up (1-month follow-up) between the two groups. Results of the linear mixed-effects models analysis are presented in Table 5. In line with hypotheses, no significant changes in outcome measures over time were observed for participants in the control group. Results indicated that participants in the HEMBP condition, compared to those in the control group, showed significant increases in mindfulness from pre- to post-test (β = 0.71, *p* = 0.001) and from pre-test to follow-up (β = 0.88, *p* < 0.001), significant increases from pre-test to follow-up in humor (β = 0.35, *p* = 0.040) and psychological well-being (β = 0.49, *p* = 0.008), and significant decreases from pre- to post-test and from pre-test to follow-up in sarcasm (β = −0.60, *p* < 0.001; β = −0.50, *p* = 0.003), cynicism (β = −0.46, *p* = 0.003; β = −0.45, *p* = 0.008), perceived stress (β = −0.71, *p* = 0.003; β = −0.51, *p* = 0.026), and gelotophobia (β = −0.41, *p* = 0.048; β = −0.49, *p* = 0.022). No significant group differences were found for fun, wit, nonsense, and satire, indicating no changes in these humor styles over time. Only irony significantly decreased from pre- to post-test in the HEMBP group compared to the control group (β = −0.45, *p* = 0.012). However, the slope difference was no longer significant after sarcasm and cynicism were added as covariates (β = −0.14, *p* = 0.412).

**Table 5:** Results from linear mixed-effects model analyses of the outcome variables by time and condition.

**Outcome**	**Intercept**			**Post-test**			**1-Month follow-up**		
**variable**	β	***p***	**95% CI**	β	***p***	**95% CI**	β	***p***	**95% CI**
Mindfulness									
WL	−0.22	0.172	[−0.54, 0.10]	−0.02	0.874	[−0.32, 0.27]	0.01	0.944	[−0.26, 0.28]
HEMBP	0.04	0.872	[−0.42, 0.49]	0.71	0.001	[0.28, 1.14]	0.88	< 0.001	[0.49, 1.27]
Fun									
WL	−0.06	0.730	[−0.43, 0.30]	0.14	0.244	[−0.10, 0.38]	0.07	0.560	[−0.17, 0.31]
HEMBP	−0.03	0.915	[−0.55, 0.50]	−0.05	0.786	[−0.39, 0.29]	0.18	0.307	[−0.17, 0.53]
Humor									
WL	−0.14	0.462	[−0.51, 0.23]	0.08	0.525	[−0.18, 0.35]	−0.15	0.204	[−0.38, 0.08]
HEMBP	0.20	0.459	[−0.33, 0.73]	0.19	0.310	[−0.18, 0.57]	0.35	0.040	[0.02, 0.68]
Nonsense									
WL	−0.08	0.647	[−0.42, 0.26]	−0.07	0.426	[−0.26, 0.11]	0.09	0.380	[−0.12, 0.31]
HEMBP	0.11	0.650	[−0.37, 0.59]	0.11	0.401	[−0.15, 0.38]	−0.09	0.577	[−0.39, 0.22]
Wit									
WL	−0.13	0.483	[−0.48, 0.23]	0.15	0.251	[−0.11, 0.42]	0.01	0.955	[−0.20, 0.22]
HEMBP	0.19	0.458	[−0.32, 0.70]	−0.15	0.421	[−0.53, 0.22]	0.04	0.820	[−0.27, 0.34]
Irony									
WL	−0.02	0.907	[−0.36, 0.32]	0.14	0.242	[−0.10, 0.39]	0.07	0.541	[−0.17, 0.31]
HEMBP	0.13	0.589	[−0.35, 0.61]	−0.45	0.012	[−0.80, −0.10]	−0.20	0.265	[−0.54, 0.15]
Satire									
WL	0.05	0.765	[−0.31, 0.41]	0.12	0.329	[−0.12, 0.35]	0.02	0.890	[−0.23, 0.27]
HEMBP	−0.15	0.574	[−0.66, 0.37]	−0.12	0.483	[−0.46, 0.22]	−0.12	0.515	[−0.48, 0.24]
Sarcasm									
WL	0.17	0.296	[−0.15, 0.49]	0.09	0.378	[−0.12, 0.30]	0.08	0.474	[−0.15, 0.31]
HEMBP	−0.16	0.499	[−0.62, 0.30]	−0.60	< 0.001	[−0.90, −0.29]	−0.50	0.003	[−0.83, −0.17]
Cynicism									
WL	0.09	0.625	[−0.27, 0.45]	0.11	0.297	[−0.10, 0.32]	0.11	0.356	[−0.12, 0.34]
HEMBP	−0.09	0.732	[−0.60, 0.42]	−0.46	0.003	[−0.76, −0.16]	−0.45	0.008	[−0.79, −0.12]
Gelotophobia									
WL	0.20	0.306	[−0.19, 0.58]	0.00	0.988	[−0.28, 0.29]	0.00	0.986	[−0.29, 0.29]
HEMBP	−0.19	0.490	[−0.74, 0.36]	−0.41	0.048	[−0.82, 0.00]	−0.49	0.022	[−0.91, −0.07]
PWB								
WL	−0.21	0.212	[−0.53, 0.12]	0.02	0.895	[−0.25, 0.28]	0.08	0.505	[−0.17, 0.33]
HEMBP	0.13	0.586	[−0.34, 0.59]	0.35	0.069	[−0.03, 0.74]	0.49	0.008	[0.13, 0.85]
Life satisfaction									
WL	−0.16	0.368	[−0.53, 0.20]	−0.01	0.967	[−0.27, 0.26]	0.07	0.581	[−0.19, 0.34]
HEMBP	0.15	0.565	[−0.37, 0.66]	0.24	0.201	[−0.13, 0.62]	0.34	0.081	[−0.04, 0.73]
Stress									
WL	0.03	0.844	[−0.31, 0.38]	0.16	0.338	[−0.17, 0.48]	−0.09	0.585	[−0.39, 0.22]
HEMBP	0.32	0.194	[−0.17, 0.81]	−0.71	0.003	[−1.18, −0.24]	−0.51	0.026	[−0.96, −0.06]

PWB, psychological well-being; WL, wait-list control; HEMBP, humor-enriched mindfulness-based program; 95% CI, 95% confidence interval. The standardized estimates for the intercept (HEMBP) indicate whether there is a significant group difference between the HEMBP and the wait-list control group at pre-test. Standardized estimates for post-test (slopes from pre- to post-test) and 1-month follow-up (slopes from pre-test to 1-month follow-up) indicate whether there is a significant time effect for the wait-list control group, and for the HEMBP whether there is a significant slope difference between the HEMBP and the wait-list control group (i.e., representing the interaction term between time and condition).

As an explorative analysis, a dose-response effect of practice time was investigated. No significant associations between average practice time during the 8-week training period and outcome change scores from pre- to post-test were found.

## 4 Discussion

Results of the randomized controlled trial provide evidence for the validity and efficacy of the HEMBP and are suggestive of the underlying theoretical framework summarized in the mindful humor filter model. Compared to individuals in a wait-list control group, participants who completed the HEMBP showed significant increases in mindfulness, psychological well-being, and benevolent humor (the latter two only from pre-test to follow-up) and significant decreases in stress, sarcasm, cynicism, and gelotophobia, and those effects were sustained up to a 1-month follow-up. Further, the feasibility and acceptability of the program were supported by good participant compliance, reflected in attendance and continued meditation practice. Attrition rates did not differ between the two groups and most of the HEMBP participants who completed the follow-up measures after 1 month indicated they engaged in ongoing formal mindfulness practice for over half the recommended amount.

The HEMBP aims to foster a mindful-humorous perspective and mindful humor. The results suggest that this aim can be achieved. A mindful-humorous perspective is at its core based on benevolent humor and is mostly mutually exclusive with sarcasm and cynicism. Consequently, and as predicted, participation in the HEMBP led to increased benevolent humor and decreased sarcasm and cynicism. There were no changes in the other humor styles except irony. Observed decreases in sarcasm and cynicism were larger in effect size than the increase in benevolent humor, perhaps indicating that it is easier to sensibilize for and inhibit these potentially hurtful forms of humor than learn an approach to humor that may be altogether new (i.e., adopting a mindful-humorous perspective and effectively using mindful humor), as this likely requires more time and practice. It is important to note that while the mindful-humorous perspective was primarily operationalized with benevolent humor, it also includes, to a lesser extent, non-harmful, virtuous, or neutral elements from other forms of humor such as fun, nonsense, and irony, that share intention and attitude with mindfulness. As such, a measure explicitly assessing a mindful-humorous perspective might result in stronger effects.

Raising participants' awareness that certain forms of humor have the potential to be hurtful, together with the strengthening of a mindful attitude, might have accounted for the decrease in gelotophobia. The HEMBP is based on learning by self-observation and experiential learning via consequences. An example of this would be observing the impact hurtful humor has on others and oneself, and whether those consequences are in alignment with one's values. During group discussions, participants often reported their intentions for using sarcasm were unconscious and that they were mostly unaware of its potential negative consequences. Among those prone to gelotophobia, this may have decreased sensitivity for interpreting humorous remarks as intentionally hurtful. Lastly, while there was a robust medium to large effect of the HEMBP on reduced stress, effects on improved well-being were less consistent, as not all well-being measures showed significant increases. This might be because it is a greater challenge to increase well-being in healthy populations with higher baseline well-being than in clinical samples (Davidson and Dahl, 2018).

The current study did not allow for a direct test of hypothesized mechanisms. Nonetheless, the results are generally supportive of the mindful humor filter model, which postulates that mindfulness might assist in the detection of incongruities, which are then selectively channeled into a mindful-humorous perspective and mindful humor. Continued mindfulness practice may have strengthened the attitudinal foundations of mindfulness and its filter function, and both might have facilitated the habitual occurrence of a mindful-humorous perspective and mindful humor while reducing potentially hurtful elements or forms of humor. These considerations are also supported by the observed decrease in irony that became non-significant once variance shared with sarcasm and cynicism was controlled for. The decrease in irony was perhaps due to its darker elements being filtered out by mindfulness.

While it can be expected that a mindfulness-based program increases mindfulness, the effects were large, which indicates that shortening practice time (e.g., compared to MBSR) and combining mindfulness and humor in a joint training does not decrease the HEMBP's impact on mindfulness. This is also indicative of synergetic effects due to shared intention, attitudes, and mechanisms. However, an active control group that includes a mindfulness-only training would be needed to confirm this. Similar effects on humor would be expected by the training of mindfulness alone, albeit to a lower extent, as has been shown by Hofmann et al. (2019), where humor as a character strength increased through mindfulness training.

In summary, the results offer empirical support for the HEMBP as a method for cultivating a mindful-humorous perspective and mindful humor, which might, in turn, initiate two positive, self-sustaining feedback loops. Internally, a mindful-humorous perspective could reinforce shared attitudinal foundations and mechanisms and as a result increase the likelihood of engaging in mindfulness practice, supported by good program adherence. Externally, mindful humor could help to foster positive emotions and relationships. The HEMBP may, therefore, effectively contribute to building healthy habits (Rothman et al., 2015).

### 4.1 Limitations and strengths

The first limitation is the comparatively small sample size of the study, which increases the probability of capitalization on chance and lowers statistical power, especially for small effects. The study was only powered for medium-sized effects so replication with a larger and representative sample is required. Second, the follow-up period of 1 month was reasonably short. Future studies should use an extended follow-up period to investigate the stability of effects over time. Third, only self-reports were employed in the study. Future studies could incorporate objective measures or other sources of data such as peer-ratings to accommodate for common method variance (Podsakoff et al., 2003). However, the focus of the HEMBP is on fostering a mindful-humorous outlook on life, and not on creating mindful humorous “stand-up comedians,” and such a perspective might elude (as mindfulness itself) a precise objective operationalization. Fourth, informal practice has been identified as important and, therefore, should be incorporated into subsequent investigations (Morgan et al., 2014). Lastly, due to rapidly increasing COVID-19 rates shortly after the HEMBP commenced, the mindfulness day session had to be canceled and classes were continued in a hybrid format. Most participants did, however, choose to continue in person, limiting the potential impact of the pandemic on training.

The current study has also several strengths. To the knowledge of the author, it was the first randomized-controlled trial evaluating the efficacy of a mindfulness-based program interfused with humor, providing evidence for its validity and efficacy. Results are suggestive of the mindful humor filter model and the HEMBP as a possibility for cultivating a mindful-humorous perspective and mindful humor, which might initiate two positive, self-sustaining feedback-loops. It could also be shown for the first time that benevolent humor, which may be particularly relevant for well-being, is malleable and can be trained together with mindfulness, while at the same time reducing sarcasm and cynicism, two forms of humor that are considered particularly harmful for intra- as well as interpersonal well-being.

### 4.2 Future research directions

Although this study contributes to both mindfulness and humor research, it also leaves several questions unanswered. Importantly, the theoretical assumptions of the mindful humor filter model underlying the HEMBP have not been tested directly. Future research could investigate whether there are indeed synergetic effects between mindfulness and humor and, if so, what the mechanisms for these effects are. Regarding the former question, a randomized controlled trial including active control groups would shed light on potential differential effects of the HEMBP compared to a mindfulness-only training such as MBSR. The latter question could be examined with dismantling studies by separating postulated mechanisms of action or using mediation analysis to investigate whether suggested mechanisms explain a significant amount of variance in observed change in outcomes.

As this study presents evidence of the efficacy of the HEMBP in promoting well-being and reducing stress among healthy adults, future research should also explore its applicability in clinical populations. The positive effects of MBPs on a broad range of outcomes and disorders in clinical contexts are well-documented (Goldberg et al., 2022). Given that the HEMBP is fundamentally a MBP rather than a humor-specific training, it appears to be well-suited for implementation in clinical settings. While there is limited research on the efficacy of humor trainings in clinical contexts, feasibility studies suggest that participation in humor trainings is associated with improvements in satisfaction with life, resilience, and cheerfulness in depressed patients (Hirsch et al., 2010; Konradt et al., 2013). Additionally, it was linked to improvements in negative and positive symptoms of schizophrenia, depression, and anxiety in patients with schizophrenia (Cai et al., 2014). Consequently, the HEMBP may be applicable to diverse patient populations.

Another question regards the possibility of shared mechanisms between mindfulness and humor. Does mindfulness share certain mechanisms with all forms of humor or are these limited to forms of humor that share intention and attitude with mindfulness. This question relates particularly to the central mechanism of mindfulness, the assumed change in perspective (reperceiving) that allows one to distance oneself from a stimulus and see it more objectively and clearly (Shapiro et al., 2006). Benevolent humor is grounded in a realistic observation of one's internal and external experiences paired with an open, accepting, conciliatory attitude (Schmidt-Hidding, 1963) facilitating a shift in perspective similar to mindfulness. In contrast, such a shift might not be possible for sarcasm and cynicism, as these arguably rely on a biased or prejudiced perspective instead. Thus, it was postulated that mindfulness shares this mechanism only with forms of humor, that share intention and attitude with mindfulness. In other words, if intention and attitude do not fit, one might be humorous, but not mindful.

## 5 Conclusion

The findings of this randomized-controlled trial empirically support the efficacy of the HEMBP for improving well-being and reducing stress. In addition, the study provides evidence for its validity, showing that both mindfulness and benevolent humor can be trained together and that doing so reduces potentially harmful or aggressive forms of humor such as sarcasm and cynicism. Thus, results are also suggestive of the validity of the mindful humor filter model that provides the theoretical basis for the HEMBP and suggest the HEMBP might be an useful extension to existing mindfulness-based programs that can foster a mindful-humorous outlook on life and promote positive emotions and relationships.

## Data availability statement

Please contact the author if you are interested in working with the data reported in this manuscript. The data described in this article are openly available on the Open Science Framework at: https://doi.org/10.17605/OSF.IO/R8VQF.

## Ethics statement

The studies involving humans were approved by the Ethics Committee of the Faculty of Arts and Social Sciences of the University of Zurich. The studies were conducted in accordance with the local legislation and institutional requirements. The participants provided their written informed consent to participate in this study.

## Author contributions

CK: Conceptualization, Formal analysis, Methodology, Writing—original draft, Writing—review & editing.
